# Knowledge and Perception of Nosocomial Infections Among Patients in a Tertiary Hospital in the Qassim Region

**DOI:** 10.3390/healthcare14162574

**Published:** 2026-08-17

**Authors:** Masaad Saeed Almutairi, Maria H. Aldawsari, Raghad S. Alharbi, Manal A. Alrasheedi, Raoom A. Almutairi, Raghad K. Almuqbel, Areej A. Aljasser, Faris S. Alnezary, Shairyar Afzal, Mohammed Almunef, Abdullah A. Alahmed, Omar A. Almohammed

**Affiliations:** 1 Department of Pharmacy Practice, College of Pharmacy, Qassim University, Buraydah 51452, Saudi Arabia; maria.aldawsari@gmail.com (M.H.A.); raghad.s.alharbii@hotmail.com (R.S.A.); m.amayer@outlook.sa (M.A.A.); raoomalmutairi@gmail.com (R.A.A.); ralmugbil@outlook.com (R.K.A.); m.almunef@qu.edu.sa (M.A.); ab.alahmed@qu.edu.sa (A.A.A.); 2 Quality Management and Safety Department, KFSH, Qassim Health Cluster, Buraydah 52366, Saudi Arabia; aaljaseer@moh.gov.sa; 3 Department of Pharmacy Practice, College of Pharmacy, Taibah University, Madinah 41477, Saudi Arabia; fnezary@taibahu.edu.sa; 4 Department of Pharmacy, DHQ Hospital Jhelum, Jhelum 49600, Pakistan; mirzashairyar@gmail.com; 5 Department of Clinical Pharmacy, College of Pharmacy, King Saud University, Riyadh 11451, Saudi Arabia; oalmohammed@ksu.edu.sa

**Keywords:** HAIs, nosocomial infection, *Acinetobacter* spp., *Pseudomonas aeruginosa*, infectious disease, public health

## Abstract

**Background:** Nosocomial infections, or healthcare-associated infections (HAIs), represent a significant global health challenge frequently associated with invasive medical procedures and extended hospitalizations. This study was designed to evaluate the current level of knowledge and the perceptions of inpatients regarding HAIs within a high-risk tertiary hospital in the Qassim region of Saudi Arabia. **Method:** A descriptive cross-sectional study was conducted from August 2024 to February 2025 at a tertiary facility in the Qassim region. The study population included 500 inpatients aged 18 years and older who consented to participate. Data were collected via individual interviews using a closed-ended questionnaire, which assessed demographics, knowledge and perceptions of HAIs on a 4-point Likert scale. **Results:** Among 500 study participants, good knowledge, fair knowledge and poor knowledge regarding HAIs were demonstrated by 57%, 38.4% and 4.6%, respectively. More than 98% of participants achieved a perception score of above 70%, indicating a profoundly positive perception level. Knowledge was significantly associated with age (*p* = 0.018), educational status (*p* < 0.01), and nationality (*p* < 0.01). Participants most commonly recognized contaminated hospital beds (95.6%), airborne transmission (91.2%), and unsterilized equipment (90%) as the potential modes of infection transmission. **Conclusions:** Sustaining ongoing infection prevention efforts and fostering improved awareness among inpatients regarding HAIs are of paramount importance in implementing effective public health prevention measures.

## 1. Introduction

Nosocomial infections, also known as healthcare-associated infections (HAIs), are one of the major issues straining health systems globally, with implications for patient and healthcare worker safety, healthcare quality, occupational hazards, and system-level economic repercussions [1,2,3]. HAIs are preventable but life-threatening and considered an adverse outcome of substandard practices in healthcare facilities, developing on or after the 3rd admission day while receiving inpatient healthcare services, and even after the patient’s discharge [4]. Poor hand hygiene, sub-optimal hygiene and sanitation of equipment and surfaces, untrained and insufficient health workers, sub-standard HAI surveillance, inadequate infection control framework, and irrational antibiotic use cultivate opportunities for pathogen transmission and reduce microbial susceptibility, leading to unintended infection dissemination from potentially antibiotic-resistant pathogens [5,6,7,8,9,10].

Common modalities of pathogen transmission are contaminated surfaces, medical devices, such as mechanical ventilators and central/urinary catheters, and healthcare personnel [9,11,12,13]. The most prevalent HAIs are bloodstream infections, pneumonia, urinary tract infections (UTIs), and surgical site infections (SSIs) [6,14]. The incidence rate is particularly high in select hospital wards, including intensive care units (ICUs), pediatric and neonatology departments, transplant units, and surgical units [5,15]. Additionally, the burden of resistant pathogens causing HAIs varies region-wise, but the most pressing microorganisms, complicating treatment modalities across the globe, encompass *E. coli*, *Staphylococcus aureus*, *vancomycin-resistant Enterococcus (VRE)*, *Clostridium difficile*, *Pseudomonas aeruginosa*, *Acinetobacter* spp., and *Klebsiella* spp. [3,4,5,15,16,17]. Care plans for HAIs are linked to significant treatment costs and additional length of stay (~11 extra days) in the hospital, leading to reappropriation of funds and resources from other crucial patient care areas [18,19,20,21].

The global death toll could reach up to 3.5 million per annum by 2050, consequent to the upward prevalence trend of HAIs, if unattended, as per recent estimates of the World Health Organization (WHO) and the Organisation for Economic Cooperation and Development (OECD) [3]. The situation is more devastating for low- and middle-income countries (LMICs) than high-income countries (HICs), where, on average, 15% of patients would acquire at least one HAI during their stay in an acute care health facility, as compared to 7% in HICs [3]. Antimicrobial resistance (AMR) further fuels this overwhelming situation; the global incidence rate is estimated to be 136 million HAIs per year, which are resistant to antibiotics [22].

The literature regarding the prevalence of HAIs in the Kingdom of Saudi Arabia (KSA) is diverse and geographically fragmented, with individual setting reports, particularly from tertiary care and large private hospitals, being quite frequent, making it difficult to sketch an overall picture [16,23,24,25]. However, the 2022 report by the Saudi Ministry of Health (MOH) from 106 MOH hospitals reported an average rate of 2.57 per 1000 central line days and 1.8 per 1000 urinary catheter days for central line-associated bloodstream infection (CLABSI) and catheter-associated urinary tract infection (CAUTI), respectively [26]. Furthermore, multiple reports suggest *Klebsiella pneumoniae* and *Acinetobacter baumannii* as the most prevalent causative agents with resistance potential for these types of infections [23,24,27,28,29]. Also, recent estimates suggest that the rate of antibiotic-resistant HAIs is approximately 20 per 1000 population per year in the KSA [22].

Nevertheless, the infection prevention and control framework is a prerequisite for accreditation with the Central Board for Accreditation of Healthcare Institutions (CBAHI), and significant improvements in quality and HAI rates have been linked to accreditation [30]. Despite this, challenges such as resource constraints, infrastructure capacity, and inadequate knowledge and training of healthcare workers hinder optimal compliance with infection prevention and control, requiring interventions such as proper infection surveillance protocols, healthcare workers’ training modules, and antimicrobial stewardship programs [31,32].

There is extensive literature on Saudi healthcare workers and medical students regarding HAIs. However, an important stakeholder, patients, lacks similar coverage within the HAI context [33,34,35,36]. Patient-to-patient or patient-to-healthcare worker transmission of infection is a major concern for HAIs [37]. Empowering patients through effective education ensures their active participation in infection prevention efforts. As HAIs can be prevented through modifiable practices such as hand hygiene, use of personal protective equipment, environmental cleaning, observing safe administration practices during invasive procedures, and timely obstruction of transmission routes, the patient’s role in these domains is clinically relevant and was inquired about accordingly in the study questionnaire. Based on the existing literature, educational status and age were likely determinants of patients’ knowledge regarding HAIs, while nationality was investigated as a context-specific factor due to potential differences in language, health literacy, and understanding of the local healthcare system. Therefore, this study aims to assess patients’ knowledge and perceptions about nosocomial infections in a high-risk tertiary care hospital in the Qassim region. Moreover, this study will identify factors influencing patients’ knowledge and perception about these infections and determine strategies to enhance care coordination and communication to reduce hospital-acquired infections and improve patient outcomes.

## 2. Materials and Methods

### 2.1. Study Design and Setting

A cross-sectional study was conducted at a tertiary hospital located in the Qassim region of Saudi Arabia. Qassim is located in the center of the Kingdom of Saudi Arabia. The region is equipped with 155 local facilities and 10 sub-provinces, providing a richly diverse patient population. According to recent estimates, the population of Qassim is approximately 1,336,179.

### 2.2. Study Population

The minimum required sample size was calculated using the single-population proportion formula for cross-sectional studies, assuming a 95% confidence level and a 5% margin of error (d = 0.05). An expected study population proportion of 50% was used because no reliable prior estimate was available. Under these assumptions, the minimum required sample size was 384 participants. To enhance the precision of the estimates and account for potential incomplete data, a total of 500 eligible inpatients were enrolled using consecutive sampling. The sample calculation is demonstrated in Table 1. The study included adult inpatients (≥18 years) admitted to a tertiary hospital in the Qassim region of Saudi Arabia between August 2024 and February 2025 who provided informed consent to participate, while patients who were critically ill or unable to provide informed consent were excluded from the study.

### 2.3. Data Collection

Data were collected using a closed-ended questionnaire conducted through individual interviews with each eligible patient. The interviews were conducted in Arabic. Participants were included only if they could understand Arabic adequately. The instrument captured demographic information and utilized specific statements to measure knowledge of HAI definitions, risk groups, transmission modes, and preventive measures. Written informed consent was obtained from all participants prior to data collection, in compliance with ethical research standards. Eligibility was determined by using predefined inclusion and exclusion criteria.

### 2.4. Questionnaire Development and Validation

This study adapts a questionnaire from a previously published paper in Nigeria that evaluates patients’ knowledge and perceptions of nosocomial infections [38]. In the Nigerian study, the instrument underwent face and content validity assessment by university lecturers in the Department of Public Health, and was pilot tested on 10 patients. The original study reported an acceptable internal consistency and a Cronbach’s alpha of 0.742. However, reliability testing was not repeated for the adapted questionnaire in the present study population, which is an obvious limitation.

### 2.5. Questionnaire Scoring

The knowledge section of the instrument comprised 25 scored subitems with options including “Yes,” “No,” and “I don’t know.” The correct option was assigned 1 point, whereas each incorrect or “I don’t know” option was assigned 0 points. The sum of all points scored for each item was presented as a total knowledge score ranging from 0 to 25. The percentage of the total score was calculated by dividing the obtained score by 25 and multiplying by 100. Scores below 50% (0–12 points) were categorized as poor knowledge, scores between 50% and 69% (13–17 points) as fair knowledge, and scores equal to or above 70% (18–25 points) as good knowledge.

For the assessment of perception, a four-point Likert scale (strongly agree = 4, agree = 3, disagree = 2, and strongly disagree = 1) was applied to a set of eight positively worded statements. The total perception score ranged between 8 and 32 and was converted into a percentage of the maximum obtainable score. Participants achieving at least 70% of the maximum score were classified as having a positive perception, while those scoring below 70% were classified as having a negative perception.

### 2.6. Statistical Analysis

Data were analyzed using Microsoft Excel® 2021 (Version 2607, Microsoft Corporation, Redmond, WA, USA) and IBM SPSS Statistics version 26 (IBM Corp., Armonk, NY, USA). Descriptive statistics were used to summarize participants’ demographic characteristics and study variables. Categorical variables are presented as frequencies and percentages. Inferential analyses were conducted to examine the associations between participants’ demographic characteristics and their categorized knowledge and perception of nosocomial infections. Pearson’s chi-square test was used to assess associations between categorical variables, while Fisher’s exact test was applied when expected cell frequencies were less than five. All statistical tests were two-sided, and a *p*-value of <0.05 was considered statistically significant.

### 2.7. Ethical Consideration

The integrity of the research process was upheld, and patient rights were secured. Through the anonymization of all data retrieved from medical records, the study assured the privacy and confidentiality of patient data. Access to the data was granted only to authorized investigators, and informed consent was obtained in accordance with the regulations in place. IRB obtained approval from local regional research ethics committee, RB board # 607/46/3791 in October 2024.

## 3. Results

### 3.1. Demographics of Study Participants and Patients’ Knowledge of Nosocomial Infections

A total of 500 participants were included in this study, of whom 52.6% (*n* = 263) were male. The most represented age group comprised individuals aged above 60 years, accounting for 31.4% (*n* = 157), followed by those aged 18–30 years, representing 20.4% (*n* = 102). The majority of participants were married, comprising 63.6% (*n* = 318), while 21.4% (*n* = 107) identified as single. Regarding educational attainment, 44.4% (*n* = 222) of participants had completed primary, intermediate, or high school education, while 22.2% (*n* = 111) held a bachelor’s degree. A smaller proportion reported having a diploma, 8.4% (*n* = 42), or post-graduate qualifications, 1.4% (*n* = 7), and 23.6% (*n* = 118) were classified as illiterate. The vast majority of participants were of Saudi nationality, comprising 86.2% (*n* = 431). This has been summarized in Table 2.

During the assessment of patients’ knowledge of nosocomial infections, 46.4% (*n* = 232) of respondents stated that they know what the term nosocomial infection means. Regarding risk groups, older people at 77% (*n* = 385) and children at 63.6% (*n* = 318) were most commonly identified, while fewer respondents considered pregnant women at 23.4% (*n* = 117), smokers at 17.8% (*n* = 89), patients in surgical wards at 22.4% (*n* = 112), and visitors at 7.4% (*n* = 37) as at-risk populations. The majority of participants, 98.2% (*n* = 491), reported poor hospital hygiene as the main risk factor for nosocomial infections. Other contributing factors identified were length of hospital stay, 66.8% (*n* = 334), and invasive non-surgical procedures, 40.4% (*n* = 202). When asked about healthcare worker behaviors that may increase the risk of hospital-acquired infections, 97.8% (*n* = 489) of patients believed that not wearing gloves while touching mucous membranes or non-intact skin posed a significant risk. Similarly, 95.6% (*n* = 478) cited failure to wear a mask during procedures or patient care activities, and 96.8% (*n* = 484) identified failure to wear both gloves and a mask during invasive procedures as risk-enhancing behaviors. In terms of preventive measures, nearly all respondents recognized effective hygiene practices: 99.4% (*n* = 497) supported thoroughly disinfecting skin and equipment, 99.2% (*n* = 496) emphasized regular handwashing, and 98.8% (*n* = 494) favored the use of protective gear. A high proportion, 89% (*n* = 445), also supported prompt replacement and removal of urinary catheters. However, fewer participants supported having patients wear goggles in surgical wards, 32.8% (*n* = 164), or restricting antibiotic use to when requested by the patient, 15.6% (*n* = 78). Concerning overall perceptions, 81.4% (*n* = 407) believed hospital-acquired infections are preventable, and 76% (*n* = 380) understood how infections spread within hospital settings. As for modes of transmission, 95.6% (*n* = 478) of respondents cited contaminated hospital beds, followed by airborne spread 91.2% (*n* = 456), unsterilized equipment 90% (*n* = 450), and improper wound dressing 73.2% (*n* = 366). Overall, the analysis of knowledge scores revealed that the majority of patients, 57% (*n* = 285), demonstrated good knowledge, while 38.4% (*n* = 192) had fair knowledge, and only 4.6% (*n* = 23) had poor knowledge. This has been summarized in Table 3.

### 3.2. Perception of Patients Towards Nosocomial Infections

**Figure 1:**
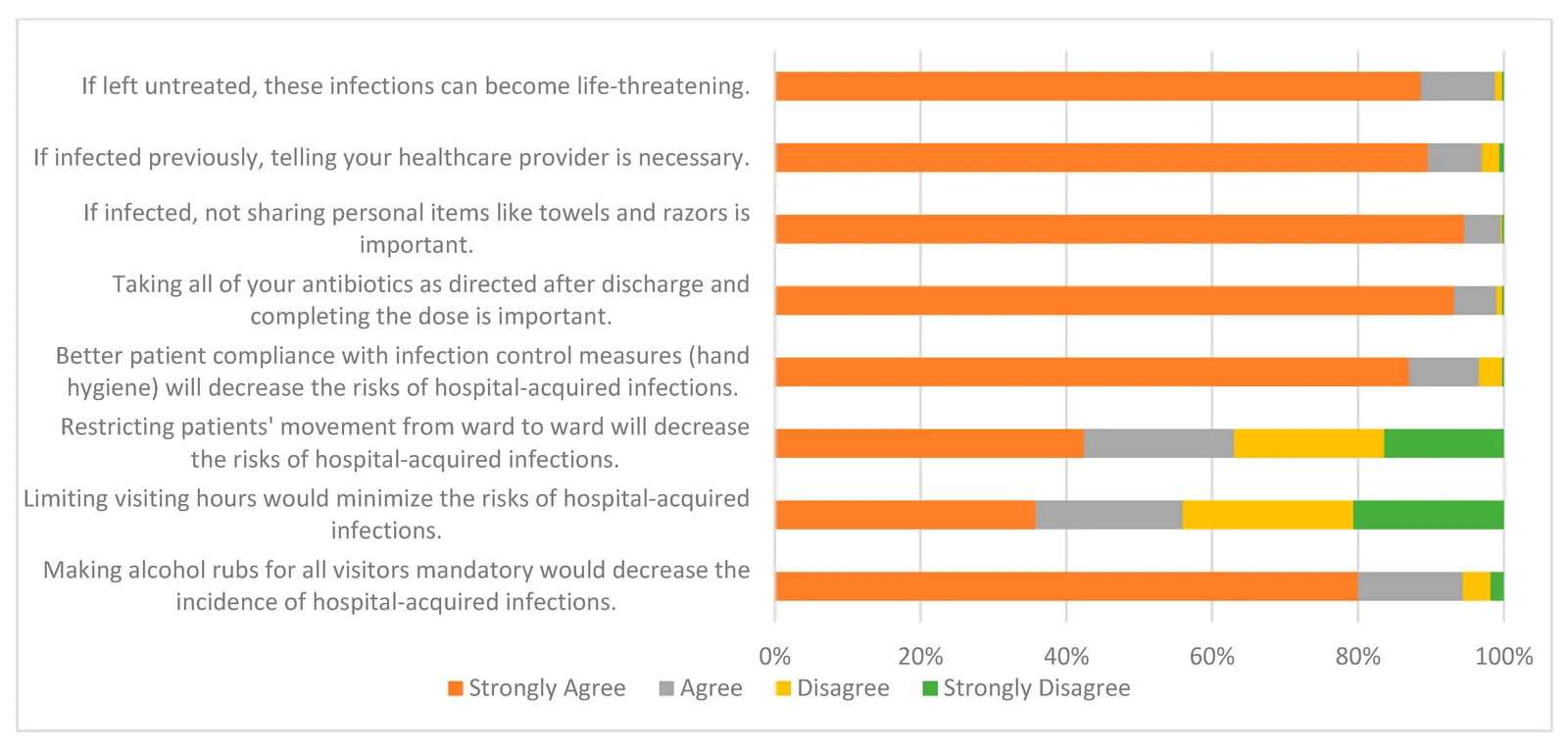
Patient’s perception of nosocomial infections. Strongly Agree—4, Agree—3, Disagree—2, Strongly Disagree—1.

Most patients (98.4% (*n* = 492)) in this study demonstrated a positive perception of nosocomial infections, as they scored above 70% on the perception scale. The mean perception score was 28.75 ± 2.61 (SD), with scores ranging from 20 to 32, indicating a generally high level of awareness and concern regarding hospital-acquired infections. More than 90% of the participants strongly agreed that not sharing personal items (towels and razors), completing a full antibiotic course, and informing healthcare providers of prior infection are the most important factors to reduce nosocomial infections. The vast majority of the participants (88.6% (*n* = 443)) strongly agreed that if left untreated, nosocomial infections can become life-threatening. Patients displayed a very high agreement on hand hygiene compliance as a prevention measure. There was noticeably more disagreement about mandatory alcohol rubs for visitors, limiting visiting hours, and restricting patient movement between wards than about the other items. This suggests patients are less convinced about the effectiveness of these institutional controls. This has been summarized in Figure 1.

### 3.3. Relationship Between Patient Demographics, Knowledge, and Perception of Nosocomial Infections

Differences in knowledge based on patient demographics, as well as the association between patient demographics and the perception of patients on nosocomial infections, were assessed in this study. There was no statistically significant difference in knowledge scores based on gender (*p* = 0.097), marital status (*p* = 0.096), or perception based on gender (*p* = 0.091) and marital status (*p* = 0.446). However, age demonstrated a statistically significant association with knowledge (*p* = 0.018), indicating that older participants demonstrated higher knowledge levels of nosocomial infections; the difference was statistically significant between participants aged above 60 and those aged 41–50 years (*p* = 0.026), with the 41–50 age group achieving higher mean knowledge scores. Details are presented in Table 4.

Educational status demonstrated a statistically significant association with patients’ knowledge of nosocomial infections (*p* < 0.01). Patients with higher educational attainment, particularly those with bachelor’s or postgraduate degrees, achieved higher mean knowledge scores compared to individuals with lower educational levels or those classified as illiterate. Conversely, no statistically significant association was found between educational status and perception (*p* = 0.102), although participants with higher education levels tended to exhibit slightly more positive perception scores. Nationality was another factor that demonstrated a statistically significant difference in knowledge scores (*p* < 0.01), with Saudi participants achieving higher mean knowledge scores compared to non-Saudi participants. However, the association between nationality and perception was not statistically significant (*p* = 0.066), although Saudi patients exhibited a slightly more positive perception overall. Age did not show a statistically significant association with perception (*p* = 0.089); however, the result suggests a potential trend, indicating that older individuals may be more aware of the consequences of nosocomial infections due to increased risk and prior healthcare experience.

## 4. Discussion

Patients are an integral part of efforts to combat HAIs [39]. Our study findings provide confidence to the healthcare managers and infection prevention team at the sampled site. Our findings indicate that participants demonstrated good knowledge, suggesting a relatively favorable level of awareness compared to previous studies, in which substandard knowledge was more prevalent [40,41]. Our estimate of 57% (*n* = 285) knowledge score is comparable to Miller and Farr et al. (62%) [42]; however, a recent comparative study by McGuckin et al. highlights that the awareness of the American consumer compared to the previous baseline (62%) has remained almost static (65%) [42,43]. Additionally, a study from South Africa reports lapses in patient awareness of post-surgery SSI, indicating a need for proper wound care counseling at discharge to ensure continuity of care [44]. Contrastingly, Madeo et al. reported that patients in the United Kingdom (U.K.) had a high level of awareness of HAIs (84%) before admission to a hospital, citing newspapers and television as primary sources of information about HAIs [45].

One important determinant of patients’ high knowledge is their own experience and encounters with HAI or with those affected in their close social circle [45,46,47]. Age was found to be statistically insignificant with respect to the perception; however, older patients in our study showed greater awareness of the consequences of HAIs. This could be linked to prior experience with an HAI. Nevertheless, the evidence varies across contexts; among healthcare workers, age is significantly associated with knowledge and/or awareness of HAIs [48,49,50]. Regarding patients, studies present contradictory evidence on the relationship between age and knowledge, with Mattner et al. [51] finding a significant association between age group and knowledge, whereas Shahab et al. [52] did not, highlighting an inconsistency in the broader evidence base regarding age as a determinant of HAI knowledge. Notably, only 46.4% of participants were aware of the technical term “nosocomial infection”. However, the overall knowledge score stands at 57%; this discrepancy can be explained by viewing the overall score as a composite value derived from many participants correctly identifying the practical features of HAI: transmission routes, risk groups, risk factors, healthcare workers’ behavior, and preventive measures. The participants who were unable to identify the technical term still achieved a good overall score.

Patients’ educational credentials empower them to collect and apply credible information in a quest to achieve the best health outcomes. In our study, a higher educational level was statistically significantly associated with the knowledge of patients with HAIs. Several other reports also complement this finding [40,41,53]. This indicates an education gap among a subset of participants who fall below the literacy scale. Research suggests that education level is also an important factor in patients’ willingness to ask healthcare providers to perform hand hygiene [54].

Hand hygiene is recognized as a vital factor in preventing infection, and patients have even reported it as a criterion when choosing a hospital or doctor. Almost all patients in our study recognized hand hygiene as an effective precautionary measure against HAIs, consistent with previous reports [55]. This provides a sound underpinning for further steps, as recent empirical evidence acknowledges patients’ active role in strengthening infection prevention efforts by asking visitors and healthcare staff to clean their hands before attending, ultimately supporting their own safety [55,56]. Engaging patients effectively and providing them with bespoke guidance would render an additional protective barrier against infections, improving outcomes for the entire patient population [56,57,58]. For example, illiterate participants (23.6%) in this study could benefit from pictorial bedside cards, short verbal counseling sessions by infection control staff with such patients, or short video/audio messages regarding hand-hygiene demonstration displayed in the waiting and inpatient areas. Similarly, for the young population, technology-driven interventional messages and social media campaigns may improve engagement. Educational material tailored to misunderstood areas, including invasive non-surgical procedures and visitor-related transmission, would improve the outcomes of the preventive campaign.

A large proportion of sampled participants correctly identified airborne transmission (91.2%), contaminated hospital beds (95.6%), and unsterilized equipment (90%) as potential modes of infection transmission, except for improper wound dressing (73.2%), suggesting an area for educational intervention. These results are quite comparable with a Pakistani study [52], except for airborne (77.4%), which was less commonly identified as an HAI transmission pathway, unlike our study. However, overall, both studies show higher knowledge scores for HAI transmission mode identification than those of Madeo et al. [45].

A reasonable proportion (81.4%) of our sampled patients believed that HAIs are preventable, which is comparable to Sahiner et al. (84.5%) [53] and Abbate et al. (88.2%) [41]. However, Shahab et al. report a lower value (62.3%). This is a sign that patients in our study understand that HAIs are not inevitable risks and are quite manageable with improved healthcare quality. Most sampled patients correctly identified older age (77%) and poor hospital hygiene (98.2%) as potential risk groups and risk factors, respectively. However, misconceptions among our study participants were widespread across different risk groups and risk factors, compared with Abbate et al., Ottum et al., and Shahab et al. Moreover, participants misidentified risk groups [pregnant women (23.4%), smokers (17.8%), and visitors (7.4%)] and risk factors [invasive non-surgical procedure (40.4%)], highlighting incomplete awareness of the risk distribution and the need for tailored information dissemination to these high-risk populations.

Our study captures a unique group in which nationality was significantly associated with knowledge. Saudi participants exhibited higher mean scores than non-Saudis; the difference probably exists due to access to health education or familiarity with the local healthcare system. Nonetheless, as we have not assessed each participant’s language proficiency or linguistic understanding in relation to the interview questions, our interpretations of this outcome should still be considered tentative. Patients in our study understood the preventive measures related to infections fairly well, with more than 90% understanding. For instance, completing antibiotic therapy after discharge and avoiding sharing razors and towels once infected are strongly endorsed by our study population, similar to what is reported by Ottum et al. [59]. However, Ottum et al. found a positive association between knowledge of preventive measures and a history of HAI. In our study, we did not estimate such an association. This lack of segregation of the patient population by prior HAI history can be considered a limitation of our study.

This study illustrates the potential effectiveness of continued efforts to promote infection prevention and public health awareness, as evidenced by higher knowledge and positive perception scores. Although knowledge gaps remain regarding at-risk groups and preventive measures, our results identify critical areas for improvement. Customized educational sessions should be arranged for those demographics with lower knowledge scores, including younger individuals, individuals with lower educational attainment, and non-Saudis.

### Limitations

There are several limitations to this study, and the results must be interpreted with care. This is a single-site study conducted in a tertiary care setting; knowledge and awareness levels may vary across healthcare settings; therefore, generalization of the findings is not recommended. Likewise, as we surveyed a large tertiary care facility, it is highly likely that the patient population at a lower-tier facility might not have sufficient knowledge and awareness scores, possibly due to less ideal efforts in line with infection prevention and educational interventions at our study setting. Secondly, this study utilizes consecutive non-probability sampling techniques at a single tertiary care facility, which again is a limitation affecting the representativeness and external validity of the findings, and subsequently the generalizability of the findings to all hospital patients or the wider population of the Qassim region. Moreover, our methodology directs direct patient interviews; we have to exclude patients who are critically ill and those unable to provide informed consent. However, we agree that the knowledge and perception metrics of this excluded cohort could differ from those of the already enrolled participants due to their experience of longer hospital stays, greater exposure to invasive procedures, and a higher risk of HAIs. Additionally, our aim in this study was never to explore the Arabic proficiency or linguistic comprehension of the interview questions posed to each participant. Therefore, the outcome related to higher Saudi Nationals’ knowledge than non-Saudis’ knowledge could be partly due to this language barrier rather than the nationality itself. As the questionnaire was adopted from a previously published study, no repeated content-validity assessment, pilot testing, or reliability analysis was conducted for this study. Therefore, contextual differences may have influenced the interpretation of the individual questionnaire item and subsequently the results offered by this study. Furthermore, cross-sectional studies cannot establish temporal precedence, so we cannot determine whether patients’ prior knowledge shaped their perceptions or whether pre-existing perceptions influenced their reported knowledge of HAIs. Additionally, the study questionnaire misses an important variable regarding the patients’ history with an HAI (personal and/or familial). Participants with a prior history of HAI will respond more cautiously to the subject and will remain extra vigilant about preventive measures, which would definitely raise the perception proportion. Assessment of this variable could significantly influence our results, as prior exposure could act as a potential confounder, potentially biasing the association between knowledge and perception. This could explain the inflated perception scores (98.4%) compared with objective participant knowledge (57%) in this study. Despite various limitations of our study, we consider it a strength that a large patient population was surveyed, resulting in a high response rate and potentially attenuating sample bias.

## 5. Conclusions

In conclusion, our study demonstrates a good knowledge and positive perception regarding HAIs among patients at a tertiary care health facility in the Qassim region. However, significant gaps remain in the less well-interpreted areas, particularly regarding invasive non-surgical procedures, visitor-related transmission, and recognition of some high-risk groups. Ongoing infection prevention practices and educational campaigns should be sustained and updated with evidence-based practices to foster a high level of awareness among inpatients, especially in identified risk groups. Moreover, future research should consider prior HAI history as an important variable in the multivariable analysis.

## Figures and Tables

**Table 1:** Sample-size calculation.

Parameter	Symbol or Formula	Assumption or Value
Key parameters to be estimated	Population proportions	Knowledge and perception of nosocomial infections among adult inpatients
Confidence level	1−α	95%
Standard normal value	$Z_{1-\alpha /2}$	1.96
Expected proportion	p	0.50
Complementary proportion	1−p	0.50
Absolute precision (margin of error)	d	0.05
Sample-size formula	$n=\frac{Z_{1-\alpha /2}^{2}p\left(1-p\right)}{d^{2}}$	—
Initial calculation	$n=\frac{1.96^{2}\times 0.50\times 0.50}{0.05^{2}}$	384.16
Minimum required sample after rounding upward	n	385
Anticipated nonresponse or incomplete data	r	20%
Adjustment formula	$n_{adjusted}=\frac{n}{1-r}$	—
Adjusted sample-size calculation	$\frac{385}{1-0.20}$	481.25
Adjusted minimum sample after rounding upward	—	482
Final enrolled participants	—	500 participants

**Table 2:** Demographics of study participants.

Variable	Number of Patients (%) (*n* = 500)
**Gender**	
Male	263 (52.6)
Female	237 (47.4)
**Marital status**	
Single	107 (21.4)
Married	318 (63.6)
Divorced	21 (4.2)
Widowed	54 (10.8)
**Age**	
18–30	102 (20.4)
31–40	65 (13)
41–50	89 (17.8)
51–60	87 (17.4)
Above 60	157 (31.4)
**Educational status**	
Primary/Intermediate/High school	222 (44.4)
Diploma	42 (8.4)
Bachelor’s degree	111 (22.2)
Post-graduate	7 (1.4)
Illiterate	118 (23.6)
**Nationality**	
Saudi	431 (86.2)
Non-Saudi	69 (13.8)

**Table 3:** Patients’ knowledge of nosocomial infection.

S/N.	Statement	Response (%)		
		Yes	No	I Don’t Know
1	**Do you know what nosocomial infection means?**	232 (46.4%)	127 (25.4%)	141 (28.2%)
2	**Which of the following groups do you believe are at risk for nosocomial infections?**			
	Older adults	385 (77%)	115 (23%)	0 (0.0%)
	Children	318 (63.6%)	182 (36.4%)	0 (0.0%)
	Pregnant women	117 (23.4%)	383 (76.6%)	0 (0.0%)
	Smokers	89 (17.8%)	411 (82.2%)	0 (0.0%)
	Visitors	37 (7.4%)	463 (92.6%)	0 (0.0%)
	Patients in surgical wards	112 (22.4%)	388 (77.6%)	0 (0.0%)
3	**What do you consider the main risk factors for nosocomial infections?**			
	Poor hospital hygiene	491 (98.2%)	9 (1.8%)	0 (0.0%)
	Invasive non-surgical procedure	202 (40.4%)	298 (59.6%)	0 (0.0%)
	Length of hospital stay	334 (66.8%)	166 (33.2%)	0 (0.0%)
4	**Which healthcare worker behaviors do you believe can increase the risk of nosocomial infections?**			
	The healthcare workers who do not wear gloves when touching mucous membranes (e.g., nose, mouth, etc.) or non-intact skin	489 (97.8%)	11 (2.2%)	0 (0.0%)
	Healthcare workers who do not wear masks during procedures and patient-care activities	478 (95.6%)	22 (4.4%)	0 (0.0%)
	Healthcare workers who do not wear gloves and mask during invasive nonsurgical procedures	484 (96.8%)	16 (3.2%)	0 (0.0%)
5	**Which of the following do you think are effective preventive measures against nosocomial infections?**			
	Thoroughly disinfecting skin and equipment	497 (99.4%)	3 (0.6%)	0 (0.0%)
	Washing hands regularly	496 (99.2%)	4 (0.8%)	0 (0.0%)
	Using protective gear such as face masks and gloves	494 (98.8%)	6 (1.2%)	0 (0.0%)
	Routinely replacing urinary catheters, and removing them promptly	445 (89%)	55 (11%)	0 (0.0%)
	Having patients wear goggles while in the surgical ward	164 (32.8%)	336 (67.2%)	0 (0.0%)
	Only prescribe antibiotics when requested by the patient	78 (15.6%)	422 (84.4%)	0 (0.0%)
6	**Do you believe all hospital-acquired infections are preventable?**	407 (81.4%)	28 (5.6%)	65 (13%)
7	**Do you understand how infections are spread in the hospital?**	380 (76%)	41 (8.2%)	79 (15.8%)
8	**What do you think are the potential modes of spread of hospital-acquired infections?**			
	Airborne	456 (91.2%)	44 (8.8%)	0 (0.0%)
	Contaminated hospital beds	478 (95.6%)	22 (4.4%)	0 (0.0%)
	Unsterilized equipment	450 (90%)	50 (10%)	0 (0.0%)
	Improper wound dressing	366 (73.2%)	134 (26.8%)	0 (0.0%)
**Score**	**Frequency (%)**	**Remark**		
<50%	23 (4.6%)	Poor knowledge		
50–69%	192 (38.4%)	Fair knowledge		
≥70%	285 (57%)	Good knowledge		
**Mean knowledge score ± SD (range): 17.646 ± 2.636 (range: 9–24)** **Total obtainable score: 25**				

**Table 4:** Overall response and the differences in the knowledge based on demographic characteristics for the participants. Association between demographics and patient’s perception of nosocomial infections.

	Knowledge					Perception		
	Poor	Fair	Good	Mean Score ± SD	*p*-Value	Negative	Positive	*p*-Value
**Overall response**	23 (4.6)	192 (38.4)	285 (57.0)			8 (1.6)	492 (98.4)	
**Gender**					0.097			0.091
Male	14 (2.8)	111 (22.2)	138 (27.6)	17.46 ± 2.57		5 (1.0)	258 (51.6)	
Female	9 (1.8)	81 (16.2)	147 (29.4)	17.85 ± 2.71		3 (0.6)	234 (46.8)	
**Marital status**					0.096			0.446
Single	5 (1.0)	43 (8.6)	59 (11.8)	17.77 ± 2.43		3 (0.6)	104 (20.8)	
Married	13 (2.6)	115 (23.0)	190 (38.0)	17.75 ± 2.63		4 (0.8)	314 (62.8)	
Divorced	0 (0.0)	9 (1.8)	12 (2.4)	17.62 ± 2.46		1 (0.2)	20 (4)	
Widowed	5 (1.0)	25 (5.0)	24 (4.8)	16.80 ± 3.06		0 (0.0)	54 (10.8)	
**Age**					0.018			0.089
18–30	7 (1.4)	40 (8.0)	55 (11)	17.49 ± 2.81		3 (0.6)	99 (19.8)	
31–40	1 (0.2)	25 (5.0)	39 (7.8)	17.98 ± 2.51		0 (0.0)	65 (13)	
41–50	1 (0.2)	31 (6.2)	57 (11.4)	18.11 ± 2.33		0 (0.0)	89 (17.8)	
51–60	3 (0.6)	27 (5.4)	57 (11.4)	18.02 ± 2.58		4 (0.8)	83 (16.6)	
Above 60	11 (2.2)	69 (13.8)	77 (15.4)	17.13 ± 2.70		1 (0.2)	156 (31.2)	
**Educational status**					<0.01			0.102
Primary/Intermediate /High school	10 (2.0)	84 (16.8)	128 (25.6)	17.74 ± 2.48		5 (1.0)	217 (43.4)	
Diploma	1 (0.2)	14 (2.8)	27 (5.4)	18.24 ± 2.45		0 (0.0)	42 (8.4)	
Bachelor’s degree	3 (0.6)	31 (6.2)	77 (15.4)	18.38 ± 2.44		0 (0.0)	111 (22.2)	
Post-graduate	0 (0.0)	1 (0.2)	6 (1.2)	19.29 ± 2.21		0 (0.0)	7 (1.4)	
Illiterate	9 (1.8)	62 (12.4)	47 (9.4)	16.47 ± 2.82		3 (0.6)	115 (23.0)	
**Nationality**					<0.01			0.066
Saudi	17 (3.4)	148 (29.6)	266 (53.2)	17.90 ± 2.57		5 (1.0)	426 (85.2)	
Non-Saudi	6 (1.2)	44 (8.8)	19 (3.8)	16.06 ± 2.55		3 (0.6)	66 (13.2)	

## Data Availability

The data that support the findings of this study are available from the corresponding authors upon reasonable request due to privacy.

## References

[1] Saleem Z., Hassali M.A., Godman B., Hashmi F.K., Saleem F. (2019). A multicenter point prevalence survey of healthcare–associated infections in Pakistan: Findings and implications. Am. J. Infect. Control.

[2] Hutton G., Chase C., Kennedy-Walker R., Hamilton H. (2024). Financial and economic costs of healthcare-associated infections in Africa. J. Hosp. Infect..

[3] WHO (2024). Global Report on Infection Prevention and Control 2024.

[4] Anand G., Lahariya R. (2025). Healthcare-associated infections. GMS Hyg. Infect. Control.

[5] Raoofi S., Pashazadeh Kan F., Rafiei S., Hosseinipalangi Z., Noorani Mejareh Z., Khani S., Abdollahi B., Talab F.S., Sanaei M., Zarabi F. (2023). Global prevalence of nosocomial infection: A systematic review and meta-analysis. PLoS ONE.

[6] Vandael E., Latour K., Goossens H., Magerman K., Drapier N., Catry B., Versporten A., Belgian Point Prevalence Survey Study Group (2020). Point prevalence survey of antimicrobial use and healthcare-associated infections in Belgian acute care hospitals: Results of the Global-PPS and ECDC-PPS 2017. Antimicrob. Resist. Infect. Control.

[7] Zingg W., Holmes A., Dettenkofer M., Goetting T., Secci F., Clack L., Allegranzi B., Magiorakos A.-P., Pittet D. (2015). Hospital organisation, management, and structure for prevention of health-care-associated infection: A systematic review and expert consensus. Lancet Infect. Dis..

[8] Storr J., Twyman A., Zingg W., Damani N., Kilpatrick C., Reilly J., Price L., Egger M., Grayson M.L., Price L. (2017). Core components for effective infection prevention and control programmes: New WHO evidence-based recommendations. Antimicrob. Resist. Infect. Control.

[9] Kramer A., Lexow F., Bludau A., Köster A.M., Misailovski M., Seifert U., Eggers M., Rutala W., Dancer S.J., Scheithauer S. (2024). How long do bacteria, fungi, protozoa, and viruses retain their replication capacity on inanimate surfaces? A systematic review examining environmental resilience versus healthcare-associated infection risk by “fomite-borne risk assessment”. Clin. Microbiol. Rev..

[10] Saleem Z. (2026). From water to wards: Status of integrated preventive strategies to break the chain of infection and antimicrobial resistance in Pakistan. Front. Pharmacol..

[11] Bennett E., VanBuren J., Holubkov R., Bratton S. (2018). Presence of Invasive Devices and Risks of Healthcare-Associated Infections and Sepsis. J. Pediatr. Intensive Care.

[12] Haque M., McKimm J., Sartelli M., Dhingra S., Labricciosa F.M., Islam S., Jahan D., Nusrat T., Chowdhury T.S., Coccolini F. (2020). Strategies to Prevent Healthcare-Associated Infections: A Narrative Overview. Risk Manag. Healthc. Policy.

[13] Rosenthal V.D., Yin R., Lu Y., Rodrigues C., Myatra S.N., Kharbanda M., Valderrama-Beltran S.L., Mehta Y., Daboor M.A., Todi S.K. (2023). The impact of healthcare-associated infections on mortality in ICU: A prospective study in Asia, Africa, Eastern Europe, Latin America, and the Middle East. Am. J. Infect. Control.

[14] Arnoldo L., Smaniotto C., Celotto D., Brunelli L., Cocconi R., Tignonsini D., Faruzzo A., Brusaferro S., Collazzo R., Mansutti M. (2019). Monitoring healthcare-associated infections and antimicrobial use at regional level through repeated point prevalence surveys: What can be learnt?. J. Hosp. Infect..

[15] Lalrinpuia B., Rai S., Kaur A., Kaur G. (2025). Antibiotic susceptibility in healthcare—Associated infections. Bioinformation.

[16] Felifel A., Soltan A., Abolseoud M.E., Eltotongy H.E., Elnagar F., Ebid M., El Shawadfy F., Eldamaty M., Abouelezz A., Saber E. (2025). Exploring the Link Between Causative Agents of Healthcare-Associated Infections and Predisposing Factors Causing Extended Hospital Stays. Cureus.

[17] Abalkhail A., Marzouk E. (2025). Healthcare-associated infections: An overview of global strategies and challenges in minimizing infection transmission. Cell Mol. Biol..

[18] De Angelis G., Murthy A., Beyersmann J., Harbarth S. (2010). Estimating the impact of healthcare-associated infections on length of stay and costs. Clin. Microbiol. Infect..

[19] Stone P.W. (2009). Economic burden of healthcare-associated infections: An American perspective. Expert Rev. Pharmacoeconomics Outcomes Res..

[20] Arefian H., Hagel S., Heublein S., Rissner F., Scherag A., Brunkhorst F.M., Baldessarini R.J., Hartmann M. (2016). Extra length of stay and costs because of health care–associated infections at a German university hospital. Am. J. Infect. Control.

[21] Tchouaket Nguemeleu E., Beogo I., Sia D., Kilpatrick K., Séguin C., Baillot A., Jabbour M., Parisien N., Robins S., Boivin S. (2020). Economic analysis of healthcare-associated infection prevention and control interventions in medical and surgical units: Systematic review using a discounting approach. J. Hosp. Infect..

[22] Balasubramanian R., Van Boeckel T.P., Carmeli Y., Cosgrove S., Laxminarayan R. (2023). Global incidence in hospital-associated infections resistant to antibiotics: An analysis of point prevalence surveys from 99 countries. PLoS Med..

[23] Alshammari I.T., Alruwaili Y. (2025). Prevalence, Microbiological Profile, and Risk Factors of Healthcare-Associated Infections in Intensive Care Units: A Retrospective Study in Aljouf, Saudi Arabia. Microorganisms.

[24] Aloufi M., Aloufi M.E., Almalki S.R., Hassanien N.S.M. (2024). Determinants of Healthcare-Associated Infections in King Abdulaziz Specialized Hospital in Taif, Saudi Arabia. Cureus.

[25] Almazeedi M.A., Al Ghadeer H.A., Bugshan A.S., Alhrthi H.L., Alshuaibi M.K., Albarqi H.H., Madkhali A.M., Maimsh O.M., A Alali S., A Al Shams A. (2023). Pattern and Frequency of Nosocomial Infections in the Pediatric Intensive Care Unit at East Jeddah General Hospital, Saudi Arabia. Cureus.

[26] Alsheddi F., Humayun T., Alsaffar M., Aldecoa Y.S., Alshammari W.H., Aldalbehi F.Z., Alanazi H., Alqahtani M., El-Saed A., Almutairi A.M. (2023). National Healthcare-Associated Infections Report 2022—Saudi Arabia. J. Infect. Public Health.

[27] Kilani M.A., Aljohar B.A., Alayed Y.A., Alshahrani N.Z., Shiha H.R., Bin Saleh G., Alshanbari N.H., Alanazi K.H. (2024). Epidemiological patterns of bacterial and fungal healthcare-associated infection outbreaks in Ministry of Health hospitals in Saudi Arabia, 2020–2021. J. Infect. Public Health.

[28] Al Dabbagh M., Alghounaim M., Almaghrabi R.H., Dbaibo G., Ghatasheh G., Ibrahim H.M., Aziz M.A., Hassanien A., Mohamed N. (2023). A Narrative Review of Healthcare-Associated Gram-Negative Infections Among Pediatric Patients in Middle Eastern Countries. Infect. Dis. Ther..

[29] Almutairi M.S., Albulaihed M., Almutairi F., Alsuhaibani Y., Alkathlan M., Alnezary F.S., Aljamhan A.S., Alresheedi A.I., Saleem Z., Almohammed O.A. (2026). Comorbidities and mortality in colistin-resistant gram-negative infections among hospitalized patients in Qassim, Saudi Arabia. Saudi Pharm. J..

[30] Alturbag M., Alyahya A. (2025). An Overview of Central Board for the Accreditation of Healthcare Institutions in Saudi Arabia: A Narrative Review. Cureus.

[31] Alslamah T., Abalkhail A. (2022). The National Strategies for and Challenges in Infection Prevention and Control of the Healthcare System in the Kingdom of Saudi Arabia (Review Study). Vaccines.

[32] Abalkhail A., Elbehiry A. (2025). Barriers and Facilitators of Health Care Workers’ Compliance with Infection Prevention and Control Practices in Health-care Facilities: A Systematic Literature Review. Indian J. Public Health.

[33] Alshammari S.A., Alrasheed S.S., Alruhaimi W.A., Albnyan A.I., Alruhaimi B., Hajj M. (2024). Knowledge, Attitude, and Practice of Standard Infection Control Precautions Among Medical Students at King Khalid University Hospital. Cureus.

[34] Alojaimy R.S., Nakamura K., Al-Sobaihi S., Tashiro Y., Watanabe N., Seino K. (2021). Infection prevention and control standards and associated factors: Case study of the level of knowledge and practices among nurses in a Saudi Arabian hospital. J. Prev. Med. Hyg..

[35] Almalki A.I., Alghamdi H.A., Tashkandy N.A. (2023). Assessment of Knowledge, Attitude, and Adherence to National Guidelines for Preventing Central Line-Associated Bloodstream Infections Among ICU Nurses of Adult Patients in Jeddah, Saudi Arabia: A Cross-Sectional Survey. Cureus.

[36] Paul E., Alzaydani Asiri I.A., Al-Hakami A., Chandramoorthy H.C., Alshehri S., Beynon C.M., Alkahtani A.M., Asiri A.H. (2020). Healthcare workers’ perspectives on healthcare-associated infections and infection control practices: A video-reflexive ethnography study in the Asir region of Saudi Arabia. Antimicrob. Resist. Infect. Control.

[37] Khan H.A., Baig F.K., Mehboob R. (2017). Nosocomial infections: Epidemiology, prevention, control and surveillance. Asian Pac. J. Trop. Biomed..

[38] Oni O., Orok E., Lawal Z., Ojo T., Oluwadare T., Bamitale T., Jaiyesimi B., Akinjisola A., Apara T. (2023). Knowledge and perception of nosocomial infections among patients in a Nigerian hospital. Sci. Rep..

[39] Knighton S., Torres-Teran M., Cadnum J., Colosimo L., Donskey C.J. (2026). A pilot study to explore the impact of just-in-time patient hand hygiene education and alcohol-based handrub use on hand contamination in outpatient settings. Am. J. Infect. Control.

[40] Merle V., Van Rossem V., Tavolacci M.-P., Czernichow P. (2005). Knowledge and opinions of surgical patients regarding nosocomial infections. J. Hosp. Infect..

[41] Abbate R., Di Giuseppe G., Marinelli P., Angelillo I.F. (2008). Patients’ knowledge, attitudes, and behavior toward hospital-associated infections in Italy. Am. J. Infect. Control.

[42] Miller P.J., Farr B.M. (1989). Survey of patients’ knowledge of nosocomial infections. Am. J. Infect. Control.

[43] McGuckin M., Storr J.A., Govednik J. (2021). Patient awareness of healthcare-associated infection risk and prevention: Has there been a change in 3 decades (1989–2019)?. Am. J. Infect. Control.

[44] Mbamalu O.N., Van Tonder E., Useh E.R., Mfeketo B., Mbengo O.P., Boutall A., Pennel T., Moloi L., Maswime S., Charani E. (2025). “Why must I get an infection, especially after surgery?” opportunities for patient engagement in infection care. Antimicrob. Steward. Healthc. Epidemiol..

[45] Madeo M., Shields L., Owen E. (2008). A pilot study to investigate patients reported knowledge, awareness, and beliefs on health care–associated infection. Am. J. Infect. Control.

[46] Anderson M., Ottum A., Zerbel S., Sethi A., Safdar N. (2013). Are hospitalized patients aware of the risks and consequences of central line-associated bloodstream infections?. Am. J. Infect. Control.

[47] Burnett E., Johnston B., Kearney N., Corlett J., MacGillivray S. (2013). Understanding factors that impact on public and patient’s risk perceptions and responses toward Clostridium difficile and other health care-associated infections: A structured literature review. Am. J. Infect. Control.

[48] Mong I., Ramoo V., Ponnampalavanar S., Chong M.C., Wan Nawawi W.N.F. (2022). Knowledge, attitude and practice in relation to catheter-associated urinary tract infection (CAUTI) prevention: A cross-sectional study. J. Clin. Nurs..

[49] Wu W., Wang W., Yuan Y., Lin L., Tan Y., Yang J., Dai L., Wang Y. (2021). Knowledge, attitude and practice concerning healthcare-associated infections among healthcare workers in Wuhan, China: Cross-sectional study. BMJ Open.

[50] Abalkhail A., Al Imam M.H., Elmosaad Y.M., Jaber M.F., Hosis K.A., Alhumaydhi F.A., Alslamah T., Alamer A., Mahmud I. (2021). Knowledge, Attitude and Practice of Standard Infection Control Precautions among Health-Care Workers in a University Hospital in Qassim, Saudi Arabia: A Cross-Sectional Survey. Int. J. Environ. Res. Public Health.

[51] Mattner F., Mattner C., Zhang I., Gastmeier P. (2006). Knowledge of nosocomial infections and multiresistant bacteria in the general population: Results of a street interview. J. Hosp. Infect..

[52] Shahab A., Shamsi T.S., Afaq E., Mustafa O., Aman D., Khan O., Zaheer A., Shaikh S., Irfan R., Saleem M. (2020). A Comparative Study: Knowledge and Practices amongst Post-Operative Patients Regarding Hospital Acquired Infections (HAI) between Private and Public Tertiary Care Setup in Pakistan. Natl. J. Health Sci..

[53] Sahiner P. (2023). What do patients know about healthcare-associated infections? What do they want to know? Ethical evaluation. Rev. Assoc. Med. Bras..

[54] Seale H., Chughtai A.A., Kaur R., Crowe P., Phillipson L., Novytska Y., Travaglia J. (2015). Ask, speak up, and be proactive: Empowering patient infection control to prevent health care–acquired infections. Am. J. Infect. Control.

[55] Wu K.-S., Lee S.S.-J., Chen J.-K., Tsai H.-C., Li C.-H., Chao H.-L., Chou H.-C., Chen Y.-J., Ke C.-M., Huang Y.-H. (2013). Hand hygiene among patients: Attitudes, perceptions, and willingness to participate. Am. J. Infect. Control.

[56] Seale H., Carlson S.J., Maley M., Clezy K., Torda A., Konecny P. (2023). Lifting the curtains of silence: Patient perceptions towards needs and responsibilities in contributing to the prevention of healthcare-associated infections and antimicrobial resistance. Am. J. Infect. Control.

[57] Hostiuc S., Molnar A.-J., Moldoveanu A., Aluas M., Moldoveanu F., Bocicor I., Dascalu M.-I., Bădilă E., Hostiuc M., Negoi I. (2018). Patient autonomy and disclosure of material information about hospital-acquired infections. Infect. Drug Resist..

[58] Anderson M., Ottum A., Zerbel S., Sethi A., Gaines M.E., Safdar N. (2013). A survey to examine patient awareness, knowledge, and perceptions regarding the risks and consequences of surgical site infections. Am. J. Infect. Control.

[59] Ottum A., Sethi A.K., Jacobs E., Zerbel S., Gaines M.E., Safdar N. (2013). Engaging patients in the prevention of health care-associated infections: A survey of patients’ awareness, knowledge, and perceptions regarding the risks and consequences of infection with methicillin-resistant Staphylococcus aureus and Clostridium difficile. Am. J. Infect. Control.

